# Distinctive Signs of Disease as Deterrents for the Endothelial Function: A Systematic Review

**DOI:** 10.3390/metabo13030430

**Published:** 2023-03-16

**Authors:** Francesco Nappi, Sanjeet Singh Avtaar Singh

**Affiliations:** 1 Department of Cardiac Surgery, Centre Cardiologique du Nord, 93200 Saint-Denis, France; 2 Department of Cardiothoracic Surgery, Royal Infirmary of Edinburgh, Edinburgh EH16 4SA, UK

**Keywords:** endothelial dysfunction, inflammation, pulmonary hypertension, diabetes, cardiovascular disease, endothelium-derived relaxing factor

## Abstract

Endothelial integrity plays a major role in homeostasis and is responsive to the numerous endogenous factors released. While its functional role in vascular tone is well described, its role in the pathophysiology of cardiovascular disease is of interest as a potential therapeutic target. We performed a systematic review to provide an overview of new therapeutic and diagnostic targets for the treatment of coronary artery disease related to endothelial dysfunction. Databases of PubMed, Ovid’s version of MEDLINE, and EMBASE were interrogated with appropriate search terms. Inclusion criteria have been met by 28 studies that were included in the final systematic review. We identified inflammation, pulmonary hypertension, diabetes mellitus and Fabry disease as pathophysiological mechanisms and explored the therapeutic options related to these conditions including medications such as Canakinumab. Endothelial dysfunction has a key role in several different pathophysiological processes which can be targeted for therapeutic options. Ongoing research should be targeted at making the transition to clinical practice. Further research is also needed on understanding the amelioration of endothelial dysfunction with the use of cardiovascular medications.

## 1. Introduction

Although studies on the endothelium function have been performed for more than 30 years, to date, no detailed guidelines on the choice of novel therapeutic and diagnostic targets have been published to deal with the onset of cardiovascular disease. This evidence collides with current clinical practice that demonstrates the important role exhibited by endogenous hyperglycemic hormones in ensuring the proper maintenance of vascular tone under pathological conditions [1,2,3]. In these circumstances the role of endothelium-derived relaxing factors is crucial and is mediated by the action of vasodilator prostaglandins, NO, and endothelium-dependent hyperpolarization (EDH) factors, as well as endothelium-derived contracting factors. Insulin levels influence synergistic coupling with endothelium-derived relaxing factors leading to the correct functioning of the macro and microcirculation. However, when the correct responses mediated by at least one of the endothelium-derived relaxing is impaired, vasoconstriction, alongside a prothrombotic and proinflammatory state is favored [4,5]. Given the importance that the endothelial function exerts in humans, an assessment is the subject of particular attention in the clinical field because if properly studied it offers the possibility of being a reliable surrogate marker of cardiovascular events. Endothelial dysfunction, when detected by impaired brachial artery flow-mediated dilatation or digital reactive hyperemia index in peripheral arterial tonometry, may be the marker of prospective cardiovascular events in patients with coronary artery disease [6,7,8]. In patients revealing a 1-SD decrease in flow-mediated dilatation or index reactive hyperemia, a duplication of risk of cardiovascular events in patients with cardiovascular disease (CVD) is markedly superimposed [9,10,11]. These considerations support the determinant role that endothelial function exerts in peripheral vascular circulation that could forecast future cardiovascular events, to the point of being a potential predictor of future cardiovascular events [9].

To encourage a broader understanding of the hallmarks of disease interfering with endothelial function and to provide guidance for clinicians, we discuss the current evidence base on the role of inflammation, diabetes mellitus, and pulmonary hypertension that may drive impaired endothelial function. Furthermore, an overview of new therapeutic and diagnostic targets for the management of coronary artery disease associated with endothelial dysfunction is provided. The purpose of this study is to understand the direct role of endothelial dysfunction in coronary artery disease. This includes the therapeutic options available that directly target the vascular endothelium and how this has ameliorated diseases alongside identifying culprit cytokines and chemokines involved in endothelial dysfunction.

## 2. Methods

### 2.1. Search Strategy

In September 2022, PubMed, Ovid’s version of MEDLINE, and EMBASE the systematic review was investigated using the terms “Endothelial vasodilation (5.202 to the present)”, “Endothelial vasoconstriction (1.102 to the present)”, “nitric oxide inflammation (3.133 to the present)”, “nitric oxide diabetes (1.117 to the present)” nitric oxide, “pulmonary hypertension (481 to the present)”, “endothelium-derived relaxing factor inflammation (3.136 to the present)”, “endothelium-derived relaxing factor diabetes (1.124 to the present)” and “endothelium-derived relaxing factor pulmonary hypertension (483 to the present)”. The search was directed to the identification of data from basic research articles and randomized controlled trials (RCT). It is important to underline that the search from the Cochrane database yielded a small number of basic research studies. On the other hand, the number of randomized studies was equal to that which emerged from the search in the other databases.

The review was registered with the OSF register of systematic reviews and followed the Preferred Reporting Items for Systematic Reviews and Meta-analyses (PRISMA) reporting guidelines. The project files are available online at (https://osf.io/tbm8y/, accessed on 15 January 2023).

### 2.2. Study Selection and Data Extraction

Searches recovered 15.778 pertinent abstracts, and after deduplication 6.928 pertinent citations were examined by 2 reviewers on their own (F.N., S.S.A.S.). The first author who performed the conceptualization (F.N.) solved the generated inconsistencies. The predefined inclusion criteria guided the review of titles and abstracts. The articles incorporated were in English and were very striking research articles based on the function of NO, EDRF, and hallmarks of disease that occurred in cases of endothelial dysfunction. Also of great relevance were the RCTs focused on novel therapeutic and diagnostic targets and observational studies on the role of inflammation, diabetes, and pulmonary. Pertinent animal studies were referred for their greater impact on the emerged role of endothelial dysfunction in supporting cardiovascular disease. Most of these were discussed in the *Arteriosclerosis, Thrombosis, and Vascular Biology (ATVB)* journal advocating a marked impact on understanding disease processes. Case reports, conference presentations, editorials, and expert opinions were excluded. A total of 390 citations were evaluated of which 28 studies met inclusion criteria and were included in the final systematic review. No significant publication bias was found, but a tendency toward higher outcome estimates in small studies was observed. The study design did not cause heterogeneity, and thus outcomes of research studies, retrospective and prospective studies were combined.

**Figure 1:**
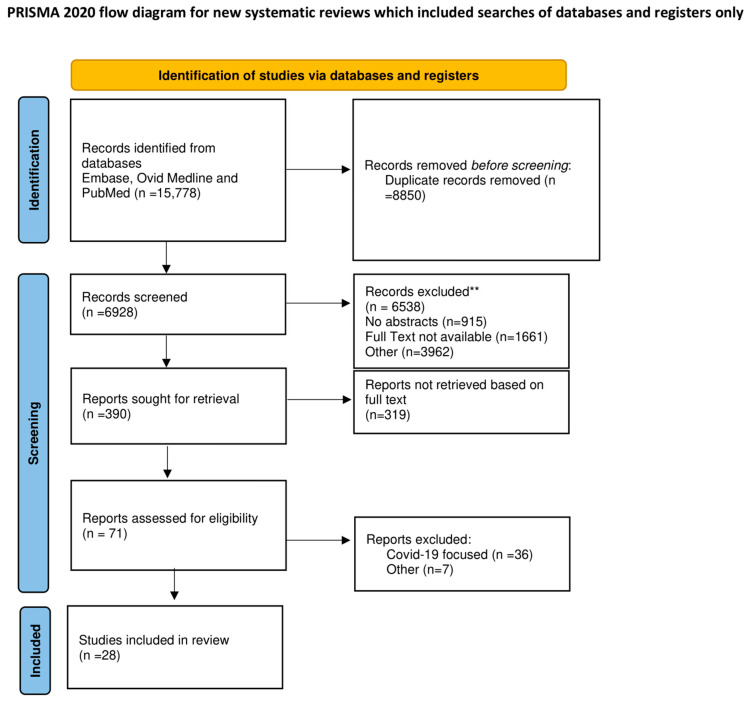
The PRISMA 2020 statement: an updated guideline for reporting systematic reviews. For more information, visit http://www.prisma-statement.org/ (accessed on 15 January 2023).

Figure 1 PRISMA Flowchart. PRISMA 2020 checklist in Supplementary Materials.

### 2.3. Endpoints and Effect Summary

The endpoints evaluated the effects of the emerging role of the hallmarks of disease that may support endothelial dysfunction focusing the analysis on studies that investigated the role of inflammation, diabetes, and pulmonary hypertension. The studies are reported alongside the discussion of results.

## 3. Results

### 3.1. Presence of Inflammation in Endothelial Dysfunction

**Figure 2:**
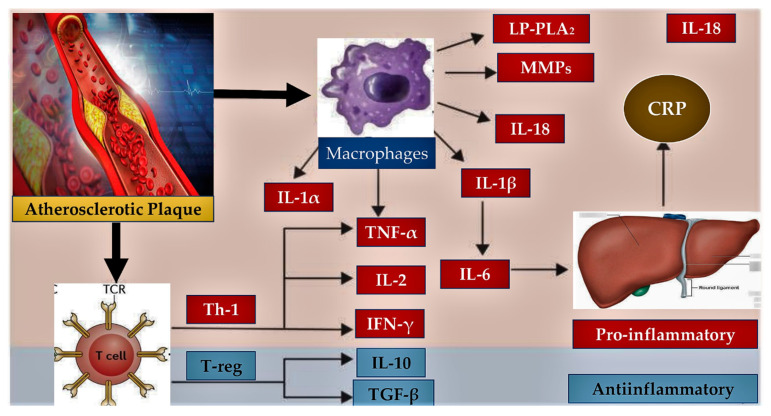
The inflammatory process entailed in atherosclerosis is represented. An intricate balance across pro-inflammatory and anti-inflammatory pathways emerged from preclinical and clinical trials. Inflammatory cells (macrophages and T-cells) and liver (CRP) regulate the balance that promotes endothelial dysfunction and the progression of atherosclerotic plaque through the production of molecules with a proinflammatory (red box) or anti-inflammatory (blue box) effect. Plaque rupture can be a consequence of the exacerbated inflammatory process. Abbreviations; CRP, C-reactive protein; MMPs, matrix metalloproteinases; IFN-γ, interferon-gamma; IL-1α, interleukin-1-alpha; IL-1β, interleukin-1-beta; IL-2, interleukin-2; IL-6, interleukin-6; IL-10, interleukin-10; IL-18, interleukin-18; Lp-PLA2, lipoprotein-associated phospholipase A2; TGF-β, transforming growth factor beta; Th-1, T-helper-1 lymphocyte; TNF-α, tumor necrosis factor alpha; T-reg, regulatory T lymphocyte.

The Inflammatory response is tightly involved in sustaining the development of endothelial dysfunction, as has been proved by the detection of IL-1β (interleukin-1β) which is an important cytokine with inflammatory function [12,13,14]. (Figure 2)

The study by Honda et al. [15] revealed a close correlation between endothelial dysfunction assessed by flow-mediated dilatation and the development of vascular inflammation. A total of 145 patients (95 men and 50 women; mean age 61.8 ± 9.5 years) underwent cardiovascular disease risk screening tests. Of these, endothelial function and vascular inflammation were investigated using flow-mediated dilatation of the brachial artery and [(18)F]-fluorodeoxyglucose positron emission tomography/computed tomography of the carotid arteries, respectively. Determination of the degree of vascular inflammation was achieved by measuring the normalized standardized uptake value in the blood, known as the target: background ratio (TBR). Absolute changes from baseline in percent FMD after 6 months of antihypertensive treatment (Δ% FMD) were investigated and whether they correlated with those in TBR in 33 drug-innocent patients with essential hypertension. Statistical significance was demonstrated by multiple logistic regression analysis in which low-density lipoprotein-cholesterol (odds ratio, 1.630 for an increase of 26 mg/dL), TBR values (odds ratio, 1.759 for an increase of 0.2), male sex (odds ratio, 0.434), and age (odds ratio, 1.767 for a 10-year increase) were substantially independently associated with percent of flow-mediated dilation in 145 patients. The report of Honda et al. initially suggested the association of vascular inflammation with endothelial dysfunction in humans disclosing the evidence that the vascular inflammation in the carotid arteries analyzed was one of the independent correlations of percent flow-mediated dilation [15].

Given the crucial role of inflammation in endothelial dysfunction, particularly in the presence of atherosclerotic plaques, the investigation has addressed the role of statins in influencing a strong anti-inflammatory effect on the atherosclerotic plaques, mainly in the course of the early phase of treatment or continuously throughout use. This strategy was considered a valid field of study. Kang et al. [16] reported findings from a prospective three-timepoint ^18^F-fluorodeoxyglucose positron emission tomography/computed tomography (^18^F-FDG PET/CT) study. Investigators evaluated the carotid artery through an assessment of the anti-inflammatory effects of statin during the early treatment period considered as the initiation of therapy to 3 months and the late treatment period relating to the period of 3 months to 1 year and their relationship with lipid and inflammatory profile changes during a year of therapy. Briefly, 20 mg/day atorvastatin was administered in nine patients with statin-naïve stable angina and with inflammatory carotid plaques, after receiving initial 18F-FDG PET/CT scanning of carotid arteries and ascending thoracic aorta. Then serial 18F-FDG PET/CT imaging was carried out for a period comprised between 3 and 12 months. The primary outcome was the inter-scan percent modification in a target-to-background ratio within the index vessel. Importantly, at 3 months of atorvastatin treatment results revealed that the mean serum low-density lipoprotein cholesterol level decreased by 36.4% to <70 mg/dL (*p* = 0.001) and the mean serum high-density lipoprotein cholesterol level raised to >40 mg/dL (*p* = 0.041). As such, both these parameters were preserved with no further lowering at up to 1 year (*p* = 0.516 and 0.715, respectively) while the mean serum high sensitivity C-reactive protein level was reduced (*p* = 0.093). The index vessel of target-to-background ratio disclosed continuous plaque inflammation lowering over 1 year, by 4.4% (*p* = 0.015) from the initiation to the 3rd month and 6.2% (*p* = 0.009) from the 3rd month to 1 year, respectively without a relationship with variations of lipid profile. The second crucial finding was disclosed by the delta target-to-background ratio of the bilateral carotid arteries and ascending aorta which decreased from 3 months to 1 year. Evidence suggested that the three-time points 18F-FDG PET/CT imaging helped prove that the anti-inflammatory effect of the statin continues throughout its use up to 1 year, albeit with the production of plasma LDL-C levels being stable below the 3-month target [16].

Four independent studies have studied the potential role exhibited by proinflammatory molecules in endothelial dysfunction that occurs in the course of diabetes and atherosclerosis [17,18,19,20]. The lack of evidence emerges from prospective human studies that have been aimed at understanding the mechanism between the beginning component of the lectin pathway, i.e., mannose-binding lectin (MBL), and endothelial dysfunction, low-grade inflammation, or carotid intima-media thickness. Furthermore, MBL-associated proteases (MASPs) and MBL-associated proteins (MAps), which mediate downstream complement activation, have not been investigated in patients who experience the development of cardiovascular disease [17,18]. Based on the previous observation that suggested the action of the lectin-complement pathway offered as molecules with a complex role in CVD, by Hertle et al. [19] in the Cohort on Diabetes and Atherosclerosis Maastricht (CODAM) Study. The prospective cohort consisted of 574 individuals (age 60 ± 7 years; 7 years follow-up), who were screened for longitudinal associations of plasma MBL, MASP-1, MASP-2, MASP-3, and MAp44 with biomarker scores reflecting low-grade inflammation and endothelial dysfunction, respectively, and carotid intima-media thickness. Another priority of the authors was to determine their association with CVD manifestation in 73 patients adjusted analysis recorded values for low-grade inflammation that was lower in the middle tertile (TMiddle) of MBL, i.e., TMiddle was 0.19 SD (0.03 to 0.34) less than TLow and 0.15 SD (−0.02 to 0.31) less than THigh. The value of carotid intima-media thickness reached 28 μm (−50 to −5) lower in patients with the greater MBL tertile (THigh) as compared to those with TMiddle and did not differ between TLow and TMiddle. Again, the evidence revealed no association between MBL and endothelial dysfunction or CAD. Furthermore, MASP-1 and MASP-2 did not show any association with any cardiovascular outcomes. In contrast, MASP-3 and MAp44 achieved, regardless of MBL levels, statistically significant values for endothelial dysfunction (for 1 SD greater MASP-3: β = 0.10 SD [0.02 to 0.18]; for 1 SD higher MAp44 β = 0.12 SD [0.04 to 0.20]), although data did not confirm the occurrence of low-grade inflammation, carotid intima-media thickness, or CVD. Evidence suggested three crucial findings. First, although MBL may concur to low carotid intima-media thickness, however, the combination of MBL with low-grade inflammation showed a non-linearity. Second, MASP-1 and MASP-2 estimates were not demonstrative of unfavorable cardiovascular results. Third, the role of MASP-3 and MAp44 in endothelial dysfunction may be established and was potentially independent of lectin-pathway activation [18].

In a recent report, Hertle et al. [20] defined the associations between the C1q pattern recognition factor and the C1-INH (C1-inhibitor) regulator and cardiovascular disease. The mandatory protocol was to evaluate cardiovascular disease events, carotid intima-media thickness, endothelial dysfunction, and low-grade inflammation. Baseline concentrations of C1q and C1-INH were determined in patients enrolled in the CODAM study (n = 574; 61% men; age, 60 ±7 years). To evaluate the 7-year incidence of CVD in participants who did not experience CVD at baseline, the investigators performed a logistic regression analysis (n = 342; 73 cases). The results reported a lower incidence of CVD that was documented in the middle tertile of C1q (T-low versus Tmiddle: odds ratio, 2.38 [95% confidence interval, 1.14–4.95]; Thigh versus Tmiddle: odds ratio, 1.96 [95% confidence interval, 0.94–4.07]). Furthermore, C1-INH values did not reach significance for the occurrence of cardiovascular disease. The data analyzed, encompassing the 7-year follow-up time, suggested that C1q and C1-INH were not, or were inconsistently linked with carotid intima-media thickness or biomarker scores and pondered the role of endothelial dysfunction and low-grade inflammation. Once again, the results offered by the CODAM study leaned towards a nonlinear association between C1q and cardiovascular disease incidence. This suggests the speculation that premature activation of the classical complement pathway may have both protective and pathological effects on human cardiovascular disease [20].

Interleukin (IL)-1β works as a cytokine with a pivotal key role in the development of CVD. An endogenous inhibitor identified as the IL-1 receptor antagonist (IL-1RA), has the function of counter-regulating IL-1β levels. This concern has been addressed in a systematic review and meta-analysis aimed to identify population-based studies on circulating IL-1RA and events related to cardiovascular disease [21,22,23].

Herder et [23] al. assessed the association between IL-1RA and incident cardiovascular disease and studied the association between IL-1RA and incident CVD along with the possible involvement of other inflammation-related biomarkers in the MONICA/KORA Augsburg case-cohort study (Multinational Monitoring of Trends and Determinants in Cardiovascular Disease/Cooperative Health Research in the Region of Augsburg). This registry included a total of 1855 cases with cardiovascular disease and 18.745 non-cases of cardiovascular disease with follow-up times between 5 and 16 years. A total of 5 cohort studies on IL-1RA and incident CVD were included in the final analysis. To reach this significant population of patients, investigators identified a relevant number of patients enrolled in the MONICA/KORA Augsburg case-cohort study that completed the cohort for the meta-analysis. The pooled standardized hazard ratio (95% confidence interval) for the incidence of cardiovascular disease was 1.11 (1.06–1.17) after adjustment for age, sex, lifestyle factors, metabolic, and anthropometric markers (*p* < 0.0001). Again, no heterogeneity in effect sizes (I2 = 0%; *p* = 0.88) was reported. Importantly, more comprehensive analyses in the MONICA/KORA trial revealed that the surfeit risk for cardiovascular disease events was mitigated by ≥10% after additional adjustment for serum levels of high-sensitivity C-reactive protein, IL-6, myeloperoxidase, soluble E-selectin, or soluble intercellular adhesion molecule-1. The observed metanalytical results from 6 population-based cohorts proved that serum IL-1RA levels were substantially linked with the risk of CVD after adjustment for multiple confounders. This combination may at least partially explain a response to triggers that promote subclinical inflammation, oxidative stress, and endothelial activation [20].

The role of inflammation as a contributory factor in all stages of the atherothrombotic process and the detection of patients with elevated inflammatory biomarkers such as high sensitivity C-reactive protein (hsCRP) may point out an increased vascular risk. The CANTOS (Canakinumab Anti-inflammatory Thrombosis Outcomes Study) RCT investigated how to direct inhibition of inflammation can reduce cardiovascular event rates [23]. Ridker et al. [24] enrolled 10,061 patients in a double-blind study using canakinumab which is a therapeutic monoclonal antibody targeting inter-leukin-1β. The study was designed to compare patients who received three doses of canakinumab (50 mg, 150 mg and 300 mg, administered subcutaneously every 3 months) and a placebo cohort. Randomization included subjects with a prior acute myocardial infarction associated with a high sensitivity C-reactive protein level of 2 mg or more per liter receiving canakinumab versus placebo. Nonfatal myocardial infarction, nonfatal stroke, or cardiovascular death constituted the primary efficacy endpoints. At 48 months, investigators recorded that the median decrease from baseline in the high-sensitivity C-reactive protein level was 26 percentage points higher in the recipients of the 50-mg dose of canakinumab, to 37 percentage points higher in patients who received the 150-mg dose of canakinumab, and 41 percentage points greater in those who received 300-mg of the drug as compared with those who were managed with placebo. Of note was that the administration of Canakinumab did not reveal a decrease in lipid levels from the baseline. Results at a median follow-up of 3.7 years, revealed that the incidence rate for the primary endpoint recorded 4.50 events per 100 person-years in the placebo arm, 4.11 events per 100 person-years in the 50-mg group, 3.86 events per 100 person-years in the 150-mg arm, and 3.90 events per 100 person-years in the 300-mg arm. The hazard ratios of patients treated with canakinumab (compared to the recipients of placebo) were 0.93 (95% confidence interval [CI], 0.80 to 1.07; *p* = 0.30) in the 50-mg cohort; 0.85 (95% CI, 0.74 to 0.98; *p* = 0.021) in the 150-mg cohort; and 0.86 (95% CI, 0.75 to 0.99; *p* = 0.031) in the 300-mg cohort. Recipients of the 150 mg dose, but not other patient-dose groups, met the prespecified multiplicity-adjusted threshold conferring statistical significance for the primary endpoint. Statistical significance was also achieved for the secondary endpoint which additionally included hospitalization for unstable angina, which caused urgent revascularization (hazard ratio vs. placebo, 0.83; 95% CI, 0.73 to 0.95; *p* = 0.005). There was a higher incidence of fatal infections with the administration of Canakinumab when compared to the placebo. There was no significant difference in all-cause mortality (hazard ratio for all canakinumab doses vs. placebo, 0.94; 95% CI, 0.83 to 1.06; *p* = 0.31). The evidence proved the efficacy of Canakinumab as anti-inflammatory therapy targeting the interleukin-1β innate immunity pathway. The drug administered at a dose of 150 mg every 3 months led to a significantly lower rate of recurrent cardiovascular events compared to the placebo, regardless of the lowering of lipid levels.

Likewise, Choi et al. [25] discussed the pathophysiology of endothelial dysfunction as drivers of early manifestation of atherosclerosis with particular regard to the part exerted by the inflammatory response and vasa vasorum role, which advocate a considerable function in promoting the pathophysiology of plaque initiation, evolution, and complications. The authors investigated the theory that vascular sections with endothelial dysfunction revealed an accumulation of macrophages and alterations at the level of vasa vasorum in subjects with prompt coronary artery disease. Optical consistency of tomography images was acquired from 40 patients who had mild coronary atherosclerosis and underwent coronary endothelial function assessment. The findings obtained included macrophages and microchannels that were examined in 76 coronaries sections corresponding to those assessed for endothelial response to acetylcholine. A change of diameter was observed in the coronary artery that manifested a response to acetylcholine. Nonetheless, this modification was further pronounced in vascular sections disclosing macrophages (−17.7 ± 14.7% vs. −6.3 ± 13.9%; *p* < 0.01) and microchannels (−15.9 ± 15.9% vs. −6.4 ± 13.5%; *p* < 0.01) than in those where cellular infiltration was lacking. Increased trends of the prevalence of generated microchannels and macrophage infiltrate associated with endothelial dysfunction, as stratified by quartiles of coronary artery diameter modification, were reported (*p* < 0.01 and *p* = 0.02 for trend, respectively). A substantial finding was those vascular sections that revealed both the presence of newly established microchannels and macrophage infiltrates (n = 12) because these conditions advocated worse endothelial function than those with macrophages alone (n = 15) and microchannels alone. (n = 15; −22.1 ± 14.6% vs. −10.9 ± 15.6% and −10.9 ± 15.6%; *p* = 0.07 and *p* = 0.06, respectively). Evidence suggested how epicardial endothelial dysfunction was linked with optical coherence tomography-identified macrophages and microchannels in mild coronary atherosclerosis advocating the crucial role of inflammation and vasa vasorum proliferation in the premature phase of coronary atherosclerosis. Table 1 shows the characteristics of the included studies relating to inflammation.

### 3.2. Pulmonary Hypertension Mediates Endothelial Dysfunction

**Figure 3:**
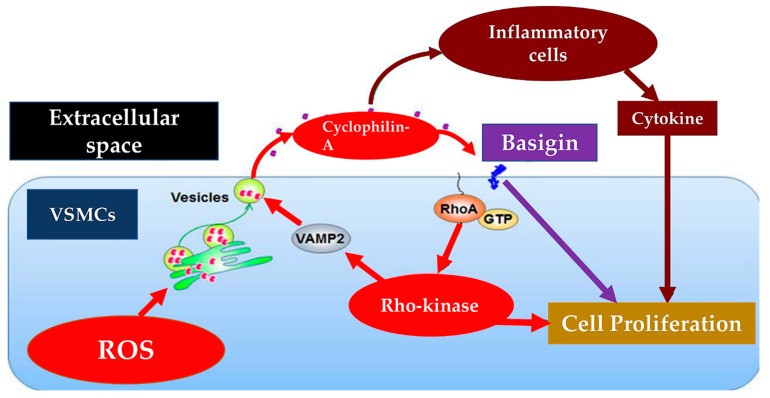
Cyclophilin A and Basigin play an important role in cell proliferation of vascular smooth muscle cells (VSMCs). In the red box and in the red arrow the mechanisms that promote activation in VSMC. Hypoxia induces the production of reactive oxygen species (ROS), which promote the secretion of cyclophilin A (CyPA).. Rho-kinase also promotes the secretion of CyPA. In the purple box, the arrow shows how extracellular CyPA recruits and stimulates inflammatory cells promoting the secretion of inflammatory cytokines. The violet box highlights the role of CyPA which directly incites the proliferation of VSMCs across Basigin, advocating additional secretion of cytokines/chemokines and growth factors. The interplay across extracellular CyPA and Basigin in VSMCs may concur with VSMC proliferation and pulmonary vascular remodeling. VAMP indicates vesicle-associated membrane protein.

The pulmonary endothelium performs multiple functions which include the control of vascular tone, blood fluidity and platelet aggregation in the pulmonary circulation. It acts as an important player in the setting of inflammation, angiogenesis and the regulation of the immunological process. Furthermore, it is an important organ with aggregated metabolic and endocrine functions. Pulmonary endothelial cells control vascular tone, and therefore local blood flow, synthesizing and delivering primary relaxing and contracting factors such as arachidonic acid metabolites via the cyclooxygenase, lipoxygenase and cytochrome P450 pathways, various peptides (endothelin, urotensin, CNP, adrenomedullin, etc.), adenosine, purines, reactive oxygen species, nitric oxide and so on. Moreover, the role promoted by endothelial ectoenzymes is a necessary step for the production of vasoactive hormones such as angiotensin II. Pulmonary endothelial dysfunction dependent on disequilibrium between the synthesis and/or release of these several endothelial factors may advocate the onset of cardiovascular pathologies such as pulmonary hypertension that may develop and perpetuate [26,27] (Figure 3).

In the context of cardiovascular pathologies, pulmonary arterial hypertension (PAH) still constitutes a life-threatening disorder with serious concern for practitioners regarding clinical management. A better understanding of the underlying mechanisms is still needed. The literature today offers a satisfactory body of knowledge to better address this pathology. The role of cyclophilin in this specific context has been widely discussed [28,29,30,31,32].

Oxidative stress and inflammation work in a substantial way to favour the development of pulmonary arterial hypertension (PAH). Oxidative stress determines a biochemical response leading to the secretion of Cyclophilin A (CypA) thus promoting inflammation and cardiovascular disease. In the pulmonary vascular environment, endothelial cell (EC) dysfunction is involved in driving the early pathogenesis of PAH [30].

Satoh et al. [28] investigated the role of extracellular CypA in PAH and differentiated the effects of acetylated CypA (AcK-CypA), which increased during oxidative stress, along with those of CypA on EC dysfunction. High levels of expressed CypA in EC specifically were observed in transgenic mice to generate a PAH phenotype. The cohort was evaluated at 3 months accounting for several changes which included increased α-smooth muscle actin expression in small arterioles, right ventricular systolic pressure, and CD45-positive cells in the lungs. Investigators undertook a mechanistic analysis with the use of cultured mouse pulmonary microvascular EC and human pulmonary microvascular EC revealing that extracellular CypA and AcK-CypA stimulated EC inflammatory signals. Results proved an enhancement in the production of VCAM1 (vascular cell adhesion molecule 1) and ICAM1 (intercellular adhesion molecule 1), degradation of IkB (nuclear factor-kappa B), and phosphorylation of p65. Increased EC apoptosis was induced by extracellular CypA and AcK-CypA and was examined by TUNEL (terminal deoxynucleotidyl transferase dUTP nick-end labeling) staining, Apo-ONE assay, and caspase 3 cleavage. The vicious circle that was generated was due to oxidative stress stimulating the secretion of CypA and AcK-CypA, further promoting the oxidative stress of EC. A difference was observed according to the effect of AcK-CypA, compared to CypA. The former induced a higher increment in oxidative stress, inflammation, and apoptosis. Using MM284 acting as a specific inhibitor of extracellular CypA, the investigators observed attenuation of EC apoptosis induced by CypA and AcK-CypA. These results revealed a substantial role of EC-derived CypA, greater than the action of AcK-CypA, that promoted the development of PAH. The presumptive mechanism involved an increased process of EC apoptosis, inflammation, and oxidative stress. The putative mechanism involved an increase in the EC apoptosis process, inflammation, and oxidative stress. The elaborated evidence has been an open window for new applicable therapies [28].

Cyclophilin A (CyPA) is a molecule produced from vascular smooth muscle cells (VSMCs) by oxidative stress and promotes VSMC proliferation. The role of extracellular CyPA and its interaction with receptor Basigin (Bsg, encoded by Bsg) has been considered in the pathogenesis of PH. Satoh et al. [29] observed that the pulmonary arteries of patients highlighting PH immunostaining disclosed robust expression of CyPA and Bsg. In addition, in a mice model, the pulmonary arteries of CyPA (±) and Bsg (±) CyPA mice displayed hypoxia for 4 weeks and recorded a considerable increment of right ventricular systolic pressure, pulmonary artery remodeling, and right ventricular hypertrophy as confronted with their littermate controls. Pulmonary VSMCs from Bsg (+/+) and Bsg (±) mice were harvested to study the role of vascular Bsg. First, investigators noted that proliferation was markedly reduced in Bsg (±) compared with Bsg (+/+) VSMCs. Second, based on the mechanistic analysis they proved that Bsg (±) VSMCs recorded a contracted extracellular signal-regulated kinase 1/2 activation and less secretion of cytokines/chemokines and growth factors such as the platelet-derived growth factor-BB. Finally, in the clinical study, patients with PH revealed augmented plasma CyPA levels in accordance with the acuteness of pulmonary vascular resistance. Furthermore, the event-free curve disclosed that the occurrence of high plasma CyPA levels was strongly predictive of poor outcomes in patients with PH. The substantial role of extracellular CyPA and vascular Bsg in the pathogenesis of PH was carefully documented [29].

Recently Rosa et al. [32] evaluated the importance of acetylation at sites K82 and K125 of two lysines that were potentially required for the release of CyPA from platelets. This process may support the potential for local delivery of CyPA thus exacerbating cardiovascular disease events. Investigators found the actual presence of CyPA in human and mouse platelets and thrombin stimulation resulted in CyPA release from platelets. However, the researchers did not find any acetylation reactions in either cell lysates or supernatants of both untreated and activated platelets. The absence of acetylation was confirmed after immunoprecipitation of CyPA from platelets. Evidence suggested that acetylation of CyPA did not result in substantial protein alteration in platelets and that secretion of CyPA by platelets could be sustained even in the absence of acetylation [32].

Pulmonary arterial hypertension is a clinical pathological entity with manifestations not confined to the lungs [33,34,35]. Several studies have reported that many signaling pathways described in PAH also play a crucial role in other diseases in which vascular remodeling occurs, which is also a pathoanatomical characterization of CAD [36,37,38]. Evidence has documented that CAD occurs 4 times more frequently in PAH than in the world population, providing a plausible reason to assert a correlation between these two diseases. Both pulmonary hypertension and coronary artery disease patients developed prolonged inflammation and a combined deranged proliferation/apoptosis process of smooth muscle cells. Meloche et al. in two landmark papers [39,40] worked on miR-223 function that reversed experimental pulmonary arterial hypertension and, in the PAH, coupled CAD mating phenotypes with a well-defined role in the increased expression of miR-223/DNA/Poly [ADP-ribose] polymerase 1/miR-204 in causing the damage. The mechanism is underpinned by activation of the miR-223/DNA/Poly [ADP-ribose] polymerase 1/miR-204 complex with subsequent overexpression of bromodomain protein 4 (BRD4) [39]. First, the restoration of the expression of miR-223 in the lungs of rats with monocrotaline-induced PAH inverted the generated PAH thus providing beneficial effects on vascular remodeling, pulmonary resistance, right ventricle hypertrophy, and survival. In the occurrence of PAH miR-223 downregulation worked substantially in numerous pathways implicated in the disease. The retrieval of its expression was effective in reversing PAH. Second, BRD4 expression in PAH was microRNA-204 dependent. The investigators through JQ1 or siBRD4 reduced the expression of BRD4 favoring a reduction of the expression of 3 major oncogenes, which are overexpressed in PAH: nuclear factor of activated T cells, B-cell lymphoma 2, and survivin. Blockage of this oncogenic signature led to a decrease in PAH-PASMC proliferation and an increase in apoptosis linked to BRD4 expression. Therefore, the mechanisms leading to pharmacological inhibition of JQ1 or molecular (siRNA) of BRD4 caused both a reversal of this pathological phenotype and the restoration of the mitochondrial membrane potential to increase the respiratory reserve capacity of the cells [39,41]. Furthermore, the investigators demonstrated that in vivo BRD4 inhibition reversed PAH induced in the Sugen/hypoxia rat model [39]. The former study of Meloche et al. implemented the evidence already reported by Archer et al. [41] that worked on the expression and activity of mitochondrial superoxide dismutase-2 (SOD2) which is recognized as the major generator of H2O2 leading to a reduction of PAH. The latter may be promoted by uncontrolled proliferation and impaired apoptosis of pulmonary artery smooth muscle cells (PASMC) contributing to vascular obstruction in patients and fawn-hooded rats (FHR) with pulmonary arterial hypertension. The findings that emerged were strongly suggestive of the tissue-specific and epigenetic role of superoxide dismutase 2. Its deficiency initiated and sustained an inherited form of pulmonary artery hypertension by impairing redox signaling and creating a proliferative, apoptosis-resistant PASMC. Finally, the increase in superoxide dismutase restored the experimental pulmonary arterial hypertension.

More recently Meloche et al. [40] hypothesized that since BRD4 also promotes triggers for calcification and remodeling processes, both important in CAD, BRD4 activation in PAH, could play a substantial role in determining the development of CAD. Investigators reported that patients with PAH distal pulmonary arteries and coronary arteries also recorded increased DNA damage, inflammation, and BRD4 overexpression. In vitro testing using human coronary artery smooth muscle cells from patients with pulmonary hypertension and coronary artery disease as well as non-PAH-non-CAD patients revealed that both pulmonary hypertension and coronary artery disease smooth muscle cells showed increased proliferation and suppressed apoptosis in a BRD4-dependent manner. In vivo tests demonstrated an improvement in PAH by the BRD4 inhibitor which was linked to a reduction in coronary remodeling with an increase in the expression level of interleukin-6. These results suggested that the increased expression of BRD4 in the coronary arteries of patients with PAH could contribute to supporting a process of vascular remodeling and the development of comorbidities [40].

Several studies demonstrated that in mouses models, hypoxia-induced mitogen factor (HIMF; also known as FIZZ1 or resistin-like molecule-β) was a potent effector for eliciting PH through triggering pulmonary vascular inflammation [42,43,44,45]. In particular, Yamaji-Kegan et al. [44], studying the initial phase of inflammation induced in a mouse model, observed that the systemic administration of HIMF could favor an increase in the apoptotic process in endothelial cells associated with higher levels in the expression of vascular inflammatory markers related to interleukin (IL)-4 production. Furthermore, HIMF, human homologous resistin (hRETN), and IL-4 were shown to activate pulmonary microvascular endothelial cells (PMVECs) by increasing angiopoietin-2 expression and inducing PMVEC apoptosis. The investigators further observed that the environment containing the hRETN-treated ECs revealed higher levels of endothelin-1 leading to marked increments in pulmonary vascular smooth muscle cell proliferation. Likewise, HIMF administration resulted in the development of PH characterized by evident pulmonary vascular remodeling with right heart failure in wild-type mice but was not observed in IL-4 knockout mice.

In a subsequent study, Johns et al. [45] demonstrated that hypoxia-inducible factor-1 (HIF-1) was a crucial downstream signaling mediator of HIMF during the development of pulmonary hypertension. In the mouse model, the degree of HIMF-induced pulmonary vascular remodeling and the development of pulmonary hypertension, both in wild-type (HIF-1α (+/+) and HIF-1α null heterozygous (HIF-1α (+/−), has been studied. HIMF-promoted pulmonary hypertension was markedly reduced in HIF-1α (+/−) mice and was driven by the activation of a dysregulated vascular endothelial growth factor receptor 2-A-vascular endothelial growth factor pathway. The work of John et al. [45] is of crucial importance because it offers a clear explanation of the interconnected mechanisms between HIMF, the production of inflammatory mediators, cellular infiltration, and pulmonary vascular remodeling. In particular, HIMF and its human homolog, the molecule resistin-β, induced a marked increase in interleukin (IL)-6 levels in macrophages and lung-resident cells by mean of a mechanism related to the activity of HIF-1α and, at least to some extent, on nuclear factor κB. Therefore HIF-1α was found to play a key role as a substantial downstream transcription factor for HIMF-induced pulmonary vascular remodeling and PH development. These data suggested the critical role of HIMF in promoting increased expression of HIF-1, vascular endothelial growth factor-A, and interleukin-6, which proved to be crucial mediators of both hypoxic inflammation and PH pathophysiology sustained by remodeling vascular [45].

Newly Lin et al. [46], in the wake of previous experience [45], studied the immunomodulatory effects of hypoxia-induced mitogenic factor (HIMF) signaling in pulmonary hypertension pathogenesis. In vivo experiments were achieved using gene-modified mice that lacked HIMF (KO [knockout]) or overexpressed HIMF human homolog resistin (hResistin). In the first step, the pro-PH role of HIMF was checked in HIMF-KO mice exposed to chronic hypoxia or Sugen/hypoxia. In the second step, based on the human in vitro and the in vivo murine model of EC, a mechanistic evaluation was performed. Activation of HIMF/hResistin that triggered the HMGB1 (high mobility group box 1) and RAGE (receptor for advanced glycation end products) pathway in pulmonary endothelial cells of both hypoxic mouse lungs in vivo and in human pulmonary microvascular ECs in vitro was evaluated. The results recorded an induction of the autophagic response, defects in BMPR2 (bone morphogenetic protein receptor 2) expression and subsequent apoptosis-resistant proliferation in human pulmonary artery smooth muscle (vascular) cells closely related to HMGB1 levels. The described effects were corroborated by analysis of endothelial cells and smooth muscle cells isolated from the pulmonary arteries of patients with idiopathic PH. Of note, both HIMF/HMGB1/RAGE-mediated autophagy and BMPR2 impairment were also recorded in pulmonary artery smooth muscle cells of hypoxic mice. Both described effects could probably be supported by a damping phenomenon of FoxO1 (forkhead box O1) exerted by HIMF. Indeed, experiments performed in transgenic mice overexpressing EC-specific hResistin supported the evidence of increased expression of EC-derived HMGB1 tempered hResistin-driven pulmonary vascular remodeling and pulmonary hypertension. Following the results observed by Lin et al., it seems evident that in HIMF-promoted pulmonary hypertension, HMGB1-RAGE signaling was critical for the crosstalk arbitration of endothelial smooth muscle cells. Evidence reported from humanized mouse models further supports the clinical implications favored by the HIMF/HMGB1 signaling axis thus suggesting that hResistin and its downstream pathway may serve as targets for the evolution of new anti-PH therapies in humans [46].

The development of chronic thromboembolic pulmonary hypertension may be considered a distinct expression of pulmonary hypertension which is due to the formation of the organized thrombotic formation of undisclosed genesis in the pulmonary arteries causing a mechanical obstruction phenomenon. The pulmonary endothelium is composed of a monolayer of endothelial cells that forms the internal cellular lining of the blood vessels represented by arteries, veins and capillaries as well as the lymphatic system. Therefore, the pulmonary endothelium is in direct contact with the blood/lymph and the circulating cells. A disorder of the endothelial function supported by coagulation dysregulation can trigger the thrombotic process. Although the endothelium exerts decisive actions in the regulation of thrombosis, hemostasis, and fibrinolysis the pathophysiology of chronic thromboembolic pulmonary hypertension persists to be poorly understood [26,27,47].

In two reports, Yaoita et al. [48,49] examined the pathophysiological and molecular mechanisms underlying chronic thromboembolic pulmonary hypertension (CTEPH) and how this fatal disease could be distinguished from pulmonary hypertension. Although CTEPH is distinct from considerable pulmonary artery obstruction related to chronic thrombus formation, it is unclear whether CTEPH is associated with a prothrombotic condition. A total of 15 patients without pulmonary hypertension (non-PAH), 19 patients with PAH and 25 patients with CTEPH were studied by measuring conventional markers, GTP-related Rap1 levels, RhoA, RalA, Rac1 and Ras in the platelets. In addition, responsiveness to ex vivo thrombin stimulation was evaluated. The results showed that the ratios of P-selectin-positive platelets in non-pulmonary hypertension patients, PAH patients, and CTEPH patients were 1.40%, 2.40%, and 2.63%, respectively (non- PH vs. CTEPH, *p* < 0.01). Second, the activated GPIIb/IIIa positive platelets reached the rate of 6.01%, 11.39% and 9.74% respectively (non-PH vs. CTEPH, *p* = 0.01). GTP-bound RhoA was 1.79%, 4.03%, and 2.01%, respectively (non-PH vs. PAH, *p* = 0.04). Moreover, RhoA bound to GTP RalA reached the rate of 1.58%, 3.02% and 2.64%, respectively (non-PH vs. PAH, *p* = 0.023; non-PH vs. CTEPH, *p* = 0.048). Regarding, Rap1, Rac1 or Ras no activation in any group was observed. Platelets from patients with CTEPH showed hyperresponsiveness to ex vivo thrombin stimulation compared with those from patients without pulmonary hypertension when surface markers were tested. The level of D-dimer or fibrin degradation product did not reveal any increase in patients with CTEPH. This study provided evidence that platelets from CTEPH patients were highly activated and showed hyperactivity to thrombin stimulation [48].

Another report by Yaoita et al. [49] investigated the role of TAFI in the involvement of CTEPH pathogenesis. A total of 68 consecutive patients were enrolled and distributed among 3 cohorts comprising 27 patients with CTEPH, 22 patients with PAH, and 19 controls without PAH, respectively. Subjects underwent right heart catheterization. A comprehensive assessment of blood clot lysis revealed that the proportion of clots remaining after 4 h was markedly greater in the CTEPH than clots retrieved from PAH patients or controls (41.9 vs. 26.5 and 24.6%, both *p* < 0.01). Furthermore, the determination of TAFI plasma levels was markedly increased in patients with CTEPH compared with those included in the PAH or control cohorts (19.4 ± 4.2 vs. 16.1 ± 4.5 or 16.3 ± 3, 3 μg/mL, both *p* < 0.05) after Interestingly, unchanged TAFI plasma levels were observed in the control group even performing percutaneous transluminal pulmonary angioplasty, which led to hemodynamic improvement. Again, clot size persisted beyond 4 h and was markedly improved by administration of CPI-2KR (an activated TAFI inhibitor) or prostaglandin E1 (an inhibitor of platelet activation). It should be noted that plasma TAFI levels were substantially correlated with the persistence of clot formation after 4 h. Finally, the range of clots persisting after 4 h was improved with the administration of an activated TAFI inhibitor. The cohorts examined revealed that plasma TAFI levels were increased in subjects with CTEPH and were related to resistance to clot lysis [49].

Table 2 reports the characteristics of the included studies relating to pulmonary hypertension.

### 3.3. Diabetes Induce Injury in Endothelium

**Figure 4:**
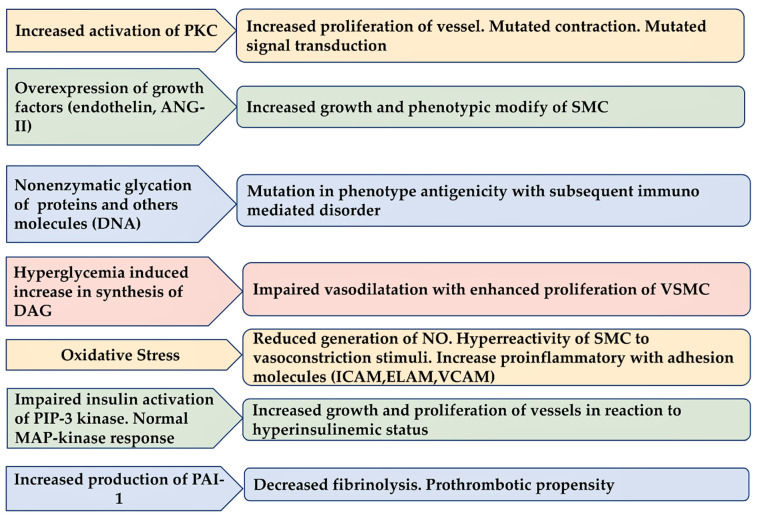
Cellular and molecular basis for endothelial dysfunction in diabetes is reported.

The effect of acute hyperglycemia on vascular function remains controversial. Several studies reported that the effect of acute hyperglycemia on endothelial and vascular smooth muscle functions across healthy and cardiometabolic diseased individuals is of relevance [1,50,51,52,53,54,55,56,57,58] (Figure 4).

A meta-analysis was drawn to compare the standardized mean difference (SMD) in endothelial and vascular smooth muscle functions between acute hyperglycemia and normoglycemia [50]. Thirty-nine articles were evaluated which included a large cohort of healthy subjects (n = 525) and patients with cardiometabolic disease (n = 540). In 39 studies, decreased endothelial function was reported (n = 1065; SMD, −1.25; 95% confidence interval, −1.52 to −0.98; *p* < 0.01), while in 6 studies investigators recorded that vascular smooth muscle function was preserved (n = 144; SMD, −0.07; 95% confidence interval, −0.30 to 0.16; *p* = 0.55) during acute hyperglycemia versus normoglycemia. However, it is important to note significant heterogeneity was observed between studies of endothelial function (*p* < 0.01). A subgroup analysis of 30 studies revealed that although the endothelial function was impaired in the macrocirculation (n = 884; SMD, −1.40; 95% confidence interval, −1.68 to −1.12; *p* < 0.01) in 9 studies analysed, it did not show reductions in microcirculation (n = 181; SMD, −0.63; 95% confidence interval, −1.36 to 0.11; *p* = 0.09).

The study of macrovascular endothelial function revealed an inverse association among age, blood pressure, and low-density lipoprotein cholesterol. In contrast, macrovascular endothelial function was firmly related to the post-occlusion interval of vascular assessment. The study suggested that macrovascular endothelial dysfunction occurred during acute hyperglycemia in both healthy and diseased subjects as opposed to microvascular dysfunction that was not revealed [50]. Recently, Lespagnol et al. [51] evaluated the extent and the contributing risk factors of early endothelial dysfunction, and the possible concomitant vascular smooth muscles (VSM) dysfunction, in subjects with type 1 diabetes using a meta-analysis. Of 58 total studies, 21 studies evaluated VSM dysfunction reporting either a standardized mean difference (SMD) (Cohen’s d) impairment of endothelial function (−0.61 (95% CI: −0.79, −0.44) that of SMD related to VSM dysfunction (−0.32 (95% CI: −0.57, −0.07). Some points deserve consideration. First, different types of stimuli were analyzed, such as occlusion-reperfusion, pharmacological substances, exercise, and heat, which did not support any impairment of the vasodilatory capacity. Second, endothelial dysfunction looked more striking within the macrovascular than in microvascular beds. The latter result showed particularly in patients with poor glycemic control [HbA1c > 67 mmol/mol (8.3%)]. The evidence from the study not only corroborated the early impairment of endothelial function, which persisted despite responses to physiological stimuli such as exercise but also highlighted VSM dysfunction in children and adults with type 1 diabetes. However, the endothelial dysfunction appeared to be more pronounced in large than small vessels, fueling the debate about their pertinent temporal aspect [51].

Loader et al. [53] assessed the vascular function during acute hyperglycemia that was induced by commercial sugar-sweetened beverage (SSB) consumption. Investigators addressed the effect on the underlying mechanisms of the nitric oxide pathway. The randomized, single-blind designed crossover study enrolled 12 healthy male participants who were scheduled to consume 600 mL (20 oz) of water or commercial SSB at 2 visits. Methods used to evaluate endothelial and vascular smooth muscle functions included laser contrast imaging coupled with iontophoresis for microcirculation while in macrocirculation evaluations were performed by brachial artery ultrasound with flow- and nitrate-mediated dilation. Impairment of microvascular and macrovascular endothelial function has been reported in subjects consuming SSB which was not manifested in those consuming water. In detail, a reduction concerning the vascular response to acetylcholine iontophoresis (208.3 ± 24.3 vs. 144.2 ± 15.7%, *p* < 0.01) and a decreased flow-mediated dilatation (0.019 ± 0.002 vs. 0.014 ± 0.002%/s, *p* < 0.01) were recorded. A substantial finding was the preservation of systemic vascular smooth muscle. Analogues changes in endothelial function were also noted in the presence of induced acute hyperglycemia in an in vivo rat model. However, endothelial function was completely restored after treatment with antioxidants of the type N-acetylcysteine and apocynin. Furthermore, it is important to underline, as revealed by ex vivo experiments, that although the generation of ROS was enhanced during acute hyperglycemia, a reduction in the bioavailability of NO in the endothelium was recorded. Instead, no modification in the activation state of endothelial nitric oxide synthase was detectable. Evidence from the RCT suggested that SSB-mediated endothelial dysfunction was in part due to increased oxidative stress that was associated with a reduction in nitric oxide bioavailability [53].

In patients with diabetes mellitus, endothelial dysfunction is a process associated with insulin resistance, inflammatory activation, and increased cardiovascular risk. Substantial evidence has emerged demonstrating the role of proinflammatory wingless-type (Wnt) family member 5a signaling via c-jun N-terminal kinase (JNK) as a crucial regulator of metabolic dysfunction, contributing to its potential relevance for vascular function [54,55]. Walther et al. [56] investigated dissimilarities within endothelial-dependent and endothelial-independent vasoreactivity. The assessment was performed both in macro- and microcirculation arterial districts in patients suffering from metabolic syndrome (MetS) with and without type 2 diabetes mellitus (T2D) and was compared with healthy counterparts. Three groups of subjects were examined to determine the existence of connections among the function of macro- and microvascular systems and abdominal adiposity, as well as inflammatory markers. The RCT enrolled 53 MetS patients without T2D and 25 with T2D who underwent cross-sectional analyses. The mandatory protocol incorporated as controls, 40-year-old, sex-matched healthy subjects, who were evaluated as regards microvascular function (cutaneous blood flow measured by laser Doppler flowmetry in response to acetylcholine and sodium nitroprusside iontophoresis), and macrovascular reactivity (flow-mediated dilatation and nitrate-mediated dilatation) together with plasma glucose, insulin, inflammatory markers and anthropometric measurements. The MetS cohort demonstrated depressed endothelial function of both micro and macrocirculatory beds compared to the control group. Patients with MetS combined with TED revealed aggravated dysfunction that involved vascular smooth muscle both in micro- and macrocirculation vascular districts compared with those with MetS but without T2D. Furthermore, upon examination of micro and macrocirculation indices, an inverse correlation between abdominal fat accumulation and inflammatory markers was reported. These results demonstrated that MetS was associated with both endothelial-dependent and endothelial-independent dysfunction and affected the micro and macro circulation. In nearly all subjects who developed diabetes mellitus, severe smooth muscle dysfunction was observed. Importantly, central abdominal fat accumulation and systemic inflammation were involved in the pathogenesis of vascular dysfunctions in MetS when they occurred in RCT participants [56].

Breton Romero et al. [57] reported the existence of enhanced activation of Wnt5a-JNK signaling that could contribute to the impairment of endothelial function in patients with diabetes mellitus. In 85 age- and sex-matched subjects, flow-mediated dilatation of the brachial artery was measured. Study groups included 42 patients with type 2 diabetes mellitus, 43 non-diabetic controls, and evaluation of human aortic endothelial cell function isolated and treated with Wnt5a. Importantly, endothelial cell function was characterized by protein expression. Briefly, endothelial cells from patients with diabetes mellitus revealed 1.3-fold higher Wnt5a levels (P = 0.01) along with 1.4-fold higher JNK activation (*p* < 0.01), without reporting a substantial difference in total JNK levels. The investigators also observed increased JNK activation that was associated with lower flow-mediated dilatation, which was broadly consistent with endothelial dysfunction (r = 0.53, *p* = 0.02). In patients with diabetes mellitus, insulin administration promoted inhibition of Wnt5a and JNK signaling while the addition of A23187 resulted in eNOS activation by enhancing nitric oxide production in endothelial cells. Concerning the endothelial cells of the non-diabetic control group, rWnt5a treatment prevented the activation of eNOS to reproduce the diabetic endothelial phenotype. In human aortic endothelial cells, the Wnt5a-induced impairment of eNOS activation and nitric oxide generation was attenuated by inhibition of Wnt5a and JNK. Results revealed that non-canonical Wnt5a signaling and JNK activity correlated with vascular reluctance to insulin action and to induce endothelial dysfunction, thus offering a novel therapeutic opportunity to protect the vasculature in patients with diabetes mellitus [57].

Cho et al. [58] evaluated whether SFRP5 was able to restore WNT5A-induced endothelial dysfunction in vitro and ex vivo. In addition, serum SFRP5 concentration was investigated for potential association with atherosclerosis in humans. The isolated thoracic aorta of a Sprague-Dawley rat model was used to induce endothelium-dependent vasorelaxation. Then, intracellular nitric oxide (NO) was measured in human endothelial cells. The protein abundance of total and phosphorylated JNK (c-Jun N-terminal kinase), AKT (protein kinase B), and endothelial NO synthase was examined in these cell lines. The study also included 282 human subjects with type 2 diabetes mellitus who were analyzed by measuring circulating levels of SFRP5 and WNT5A and brachial-ankle pulse wave velocity. In the rat thoracic aorta, the results showed that SFRP5 dose-dependently restored the impaired Wnt5-induced vasodilation via an endothelial NO synthase-dependent mechanism. Of note, in human endothelial cells, it was observed that the WNT5A-induced decrease in NO production via endothelial NO synthase was reversed following SFRP5 treatment. Two results were important. The first result showed that the WNT5A-induced changes in the phosphorylation of JNK, AKT and endothelial NO synthase were improved by the administration of SFRP5. In the second data, it emerged that in the human population with type 2 diabetes mellitus, the serum concentration of SFRP5 was strongly consistent with the brachial-ankle pulse wave velocity (r = 0.146; *p* = 0.024). Multivariate linear regression analysis also demonstrated that serum SFRP5 concentration was independently associated with brachial-ankle pulse wave velocity after adjustment for potential confounders [B (SE) = 7.40 (3.35); *p* = 0.028]. These results suggested the potential compensatory role exerted by SFRP5 against atherosclerosis in a state of metabolic dysfunction [58].

We learned that in humans’ high glucose level concentrations acutely lead to endothelial cell oxidative stress and this process is suggested to trigger diabetes-related macro- and microvascular disorders. Although numerous clinical studies performed in healthy humans have established that acute hyperglycemia, sustained by mixed meals or oral glucose, could reduce arterial vascular function; however, diet influences autonomic production, insulin secretion, incretin secretion, and blood pressure. All of these factors could independently alter vascular function and obscure the effect of acute hyperglycemia per se. Given this evidence, surprisingly, the literature lacks a body of studies evaluating the effect of acute hyperglycemia on both macro and microvascular function during the control of plasma insulin concentrations. Based on this evidence, Horton et al. [52] designed an RCT to compare macrovascular and microvascular functional responses to euglycemia and hyperglycemia. In detail, in healthy young adults’ functional responses such as brachial artery flow-mediated dilatation, carotid-femoral pulse wave velocity, and post-ischemic brachial artery flow velocity cardiac and skeletal muscle perfusion by ultrasound with medium contrast agents after octreotide infusion in both protocols to prevent endogenous insulin release were investigated. Of note, the acute hyperglycemia determined by intravenous glucose infusion ameliorated flow-mediated dilation of the brachial artery (*p* = 0.004), increased skeletal muscle microvascular blood volume and blood flow (*p* = 0.001), and heart muscle microvascular blood volume expansion (*p* = 0.014). In addition, the investigators reported no changes in measures of vascular function during euglycemia sustained by octreotide administration. The evidence demonstrated in the randomized study was a substantial difference between acute hyperglycemia induced by the meal and that induced by intravenous glucose infusion over 4 h. The latter improved flow-mediated dilatation of the brachial artery, stimulated the microvascular function of cardiac and skeletal muscle and did not compromise aortic stiffness. This study opposes different results compared to previous ones reported on acute vascular dysfunction of the great arteries during oral ingestion of glucose or mixed meals which may be favored by marked differences in the analyzed populations and by the presence of meal-induced humoral or neural factors in addition to hyperglycemia itself [52].

Table 3 highlights the included studies relating to diabetes.

### 3.4. Endothelial Dysfunction and Fabry Disease

Evidence reported a specific role of Globotriaosylceramide (Gb3) in inducing KCa3.1 downregulation in patients who experienced Fabry disease (FD) [59,60]. Choi et al. [61] studied KCa3.1 endocytosis and degradation caused by Gb3. The two cohorts used had KCa3 downregulation, mainly plasma membrane-localized KCa3.1, in both Gb3-treated mouse aortic endothelial cells (MAEC) and human umbilical vein endothelial cells. Gb3-induced KCa3.1 downregulation was avoided by lysosomal inhibitors but not by a proteasomal inhibitor. Agents causing endoplasmic reticulum stress were not responsible for causing the downregulation of KCa3.1. Gb3 had the effect of upregulating the protein levels of early endosome antigen 1 and lysosome-associated membrane protein 2 in MAECs. In an animal model of FD reproduced in aged α-galactosidase A (Gla) knockout mice, with downregulation of KCa3.1, early endosome antigen 1 and lysosome-associated membrane protein 2 expression was reported. In contrast, no fundamental difference was recorded in the exhibition of early endosome antigen 1 and lysosome-associated protein 2 between Gla-knockout juveniles and wild-type MAEC. The significant data observed demonstrated that in aged Gla-knockout MAECs, clathrin was translocated near the cell border and clathrin knockdown recovered KCa3.1 expression. This effect was enhanced by Rab5, an effector of early endosome antigen 1, which was upregulated, and the knockdown of Rab5 restored KCa3.1 expression, current, and endothelium-dependent relaxation. Evidence suggested an acceleration of the endocytosis and lysosomal degradation phenomena of endothelial KCa3.1 that induced by Gb3 and via a clathrin-dependent process, leading to endothelial dysfunction in FD [61].

Importantly, Fabry disease also promotes cardiac involvement. Although it is current from the beginning of life, however, the clinical manifestations are detectable starting from the third or fourth decade. Subsequently, heart involvement evolves into forms of severe disease advocating marked substantial morbidity. Premature death is due to heart failure, arrhythmia and stroke. In Fabry disease, the most common structural cardiac disorder observed is left ventricular hypertrophy which is supported by the presence of cardiac symptoms such as arrhythmias and dyspnea [62]. Ventricular hypertrophy increases with age with a predilection for males over males. female concerning clinical manifestations. Subjects who experience an increase in cardiac mass have proportional renal dysfunction [62]. The heart valves may be involved in AFD with an accumulation of Globotriaosylceramide in the structure of the valve leaflets. Secondary fibrosis and calcification may advocate a valvular dysfunction [63]. Although contemporary studies have shown that mild regurgitation involves the aortic, mitral and tricuspid valves, however, the most frequent lesions are recorded in patients with advanced disease [64]. Valve disease was reported in 14.6% of patients entering the Fabry outcome survey registry albeit with little progress towards the need for surgery, suggesting that the lesions were rarely of hemodynamic significance [62]. However, in conjunction with coronary artery disease [62,65,66,67], the valvular disorder can worsen, manifesting itself with the characteristics of a mixed degenerative-ischemic disorder. These patients underwent a combined intervention directed at coronary and valvular disease; the latter was treated at the valvular and subvalvular levels [68,69,70,71,72].

## 4. Conclusions

Endothelial dysfunction plays a substantial role in multiple pathophysiological processes that can be targeted for therapeutic options. Ongoing research should aim at the transition to clinical practice. There is a significant overlap between the use of cardiovascular medications and their effects on the endothelium. Further research is also needed on understanding the amelioration of endothelial dysfunction with the use of cardiovascular medications which may explain some of the prognostic benefits that reduce both morbidity and mortality. Future directions should include further primary research on the role of endothelial dysfunction as a pathophysiological mechanism for cardiovascular diseases.

## Figures and Tables

**Table 1:** Characteristics of the Included Studies.

First Author/Year Ref	Type of Study	Cohort	Aims	Finding
Honda et al. (2016) Arterioscler Thromb Vasc Biol [15]	Human Prospective Single Center (USA)	145 pts (95 men and 50 women)	To evaluate the relationship between endothelial function and vascular inflammation by mean of flow-mediated dilation (FMD) and ^18^FDG-PET	Vascular inflammation in the carotid arteries evaluated independently correlates to decreased %FMD suggesting the association of vascular inflammation with endothelial dysfunction
Kang et al. (2019) Front Immunol [16]	Prospective Single Center (Korea)	Nine statin-naïve SA patients with inflammatory carotid plaques	To evaluate anti-inflammatory effects of statin initiation to 3 and 3 months to 1 year) by mean o 18F-FDG PET/CT	The anti-inflammatory effect of the statin continues to maintain an effect for up to 1 year. However, stable plasma LDL-C levels below the 3-month target were produced.
Krog et al. (2017) Clin Exp Immunol [18]	Human/Animal model Prospective Single Center (Denmark)	100 pts with type 2 diabetes vs. 100 sex- and age-matched controls Streptozotocin-induced diabetes mouse	To study SNPs in the MASP1 gene and altered MASP-1, MASP-3 and MAp44	Higher levels of MASP-1 levels among pts with type 2 diabetes and diabetic mice
Hertle et al. (2016) Arterioscler Thromb Vasc Biol [19]	Prospective Multicenter (Netherlands, Denmark) CODAM study (Cohort on Diabetes and Atherosclerosis Maastricht)	574 pts cIMT 73 pts CVD	To study MBL-associated proteases (MASPs) and MBL-associated proteins (MAps) in complement activation and CVD	High MBL may contribute to low cIMT. MASP-1 and MASP-2 were not associated with adverse cardiovascular outcomes. MASP-3 and MAp44 crucial role in endothelial dysfunction
Hertle et al. (2018) Arterioscler Thromb Vasc Biol [20]	Prospective Multicenter (Netherlands Denmark) CODAM study (Cohort on Diabetes and Atherosclerosis Maastricht)	574 pts cIMT 73 pts CVD	To determine the associations between factor C1q its regulator C1-INH and CVD	Nonlinear association between C1q and incident CVD
Herder et al. (2017) Cardiovasc Diabetol. [22]	Prospective Single Center (Germany)	1107 pts KORA F4 study.	Whether higher IL-22 levels are associated with lower diabetes incidence.	High serum levels of IL-22 were inversely associated with cardiometabolic risk factors. these associations did not translate into an increased risk for type 2 diabetes
Herder et al. (2017) Arterioscler Thromb Vasc Biol [23]	Meta-Analysis Single Center (Germany)	5 cohort studies IL-1RA MONICA/KORA Augsburg case-cohort study 1855 pts CVD 18,745 noncases CVD	To evaluate circulating IL-1RA and the incidence of CVD	Serum IL-1RA levels were associated with the risk of CVD after adjustment for multiple confounders. IL-1RA lead to subclinical inflammation, oxidative stress, and endothelial activation.
Ridker et al. (2017) NEJM J [24]	RCT Multicenter Center CANTOS Trial	10,061 patients canakinumab (50 mg, 150 mg, and 300 mg) 3344 placebo group	To compare the therapeutic effect of a monoclonal antibody targeting interleukin-1β	Canakinumab at a dose of 150 mg every 3 months was effective as an anti-inflammatory against interleukin-1β leading to a significantly lower rate of recurrent cardiovascular events than placebo,
Choi et al. (2014) Arterioscler Thromb Vasc Biol [25]	Multicenter Center (USA/Korea)	40 pts with mild coronary atherosclerosis	To study endothelial dysfunction in pts with early CAD presenting macrophages and vasa vasorum infiltrates.	Epicardial endothelial dysfunction was associated with optical coherence tomography -which identified macrophages and microchannels in mild coronary atherosclerosis.

Abbreviations; cIMT, carotid intima-media thickness; C, complement; C1-INH, C1-inhibitor; CAD, CVD, cardiovascular disease; ^18^FDG-PET, [^18^F]-fluorodeoxyglucose-positron emission tomography; FMD, flow-mediated dilation; KORA, Kooperative Gesundheitsforschung in der Region Augsburg; MAp44; mannan-binding lectin-associated protein of 44 kDa; Map, MBL-associated protein; MASP, MBL-associated serin protease; MBL, mannose-binding lectin; SA, stable angina; pts, patients; SNPs, nucleotide polymorphism

**Table 2:** Characteristics of the Included Studies.

First Author/Year Ref	Type of Study	Cohort	Aims	Finding
Satoh et al. (2008) Antioxid Redox Signal [28]	Animal Model Single Center (Japan)	CyPA knockout mice vs. Wild-type mice vs. VSMC-Tg mice	To evaluate contribute of CyPA to vascular remodeling	CyPA regulates inflammatory cell accumulation, flow-mediated vascular remodeling and intima formation.
Satoh et al. (2014) Circ Res [29]	Animal Model Single Center (Japan)	CyPA (±) mice vs. Bsg (±) mice	To determine the role of CyPA/Bsg signaling in the development of PH.	Increased CyPA levels in patients with PH. Cell proliferation was reduced in Bsg (±) compared with Bsg (+/+) VSMCs.
Xe et al.(2017) Arterioscler Thromb Vasc Biol [30]	Human/Animal Model Single Center (USA)	CyPA (±) mice vs. Human pulmonary EC	To evaluate the role of extracellular CypA in PH. To compare the effects of acetylated CypA (AcK-CypA) and CypA on EC dysfunction.	EC-derived CypA (especially AcK-CypA) favor PH due to apoptosis, inflammation, and oxidative stress
Rosa et al. (2022) Int J Mol Sci. [32]	Animal/Human Model Prospective Multicenter (Germany, UK,)	CyPA (±) mice vs. Human pulmonary EC	Whether K82 and K125 acetylation is required for the release of CyPA from platelets	Acetylation of CyPA no major protein modification in platelets. CyPA acetylation is not required
Meloche et al. (2014) Circulation [39]	Prospective Single Center (Canada)	Human PH EC vs. Healthy tissues/cells	To study PAH-PASMCs increasing during activation of poly (ADP-ribose) polymerase-1 (PARP-1)	PH development related to DNA damage/PARP-1 signaling pathway
Meloche et al. (2015) Am J Physiol Cell Physiol [40]	Prospective Single Center (Canada)	Human PH EC vs. Healthy tissues/cells	Wether miR-223 downregulation triggers PARP-1 overexpression	Downregulation of miR-223 in PH
Archer et al. (2010) Circulation [41]	Animal/ Human Model Single center (USA)	FHR (PH rat) vs. * Sprague-Dawley rat PH pts	To study the expression of SOD2 and its correlation with PH	Epigenetic SOD2 deficiency induces PH due to impairing redox signaling and creating a proliferative, apoptosis-resistant PASMC
Yamaji-Kegan et al. (2014) Am J Physiol Lung Cell Mol Physiol [44]	AnimalModel Single Center (USA)	IL-4/STAT6 KO mice vs. wild-type (WT) mice	To evaluate how HIMF lead to lung inflammation and vascular remodeling	IL-4 signaling exerts a substantial role in HIMF-induced lung inflammation and vascular remodeling.
Johns et al. (2016) Arterioscler Thromb Vasc Biol [45]	Animal Model Single Center (USA)	HIF-1α(+/−) mice vs. wild-type (HIF-1α (+/+) vs. Human resistin-like molecule-β	To evaluate hypoxia-inducible factor-1 (HIF-1) is a critical downstream signal mediator of HIMF during PH development.	HIMF can induce HIF-1, vascular endothelial growth factor-A, and interleukin-6. Mediators for hypoxic inflammation and PH pathophysiology.
Lin et al. (2018) Arterioscler Thromb Vasc Biol [46]	Animal/Human Model Single Center (USA)	HIMF KO mice vs. Human RELM-β	To investigate the immunomodulatory properties of HIMF signaling in PH pathogenesis	In HIMF-induced PH, HMGB1-RAGE mediates EC-smooth muscle cell crosstalk

Abbreviations; AcK-CypA, CypA; Basigin; Cyclopillin A; EC, Endothelial cell; FHR, Fawn-hooded rat; PARP-1, poly (ADP-ribose) polymerase-1; h-restin; homolog resistin; HIF-1, hypoxia-inducible factor-1; HIF-1α (+/−), HIF-1α heterozygous null; HIMF, Hypoxia-induced mitogenic factor; HMGB1, high mobility group box 1; KO, knockout; PASMC, pulmonary arterial smooth muscle cell; PH, pulmonary hypertension; Pt, patient; RAGE, receptor for advanced glycation end products; RELM, Resistin-like molecule; SOD2, mitochondrial superoxide dismutase-2; VSMC, smooth muscle cell; VSMC-Tg, overexpress CyPA; WT, wild-type. * Sprague-Dawley rat selective for PASMCs; HIMF or FIZZ1 (found in inflammatory zone-1) or RELM (resistin-like molecule-α).

**Table 3:** Characteristics of the Included Studies.

First Author/Year Ref	Type of Study	Cohort	Aims	Finding
Loader et al. (2015) Arterioscler Thromb Vasc Biol [50]	Human Study level meta-analysis	525 healthy pts 540 cardiometabolic pts	To compare acute hyperglycemia in EF and VSMF	In healthy and diseased subjects macrovascular but not microvascular endothelial dysfunction during acute hyperglycemia was revealed
Lespagnol et al. (2020) Front Endocrinol. [51]	Human Study level meta-analysis	21 study	To evaluate early EF and VSMF alteration in type 1 diabetes.	In children and adults, VSM dysfunction with type 1 diabetes is demonstrated. Endothelial dysfunction s more pronounced in large than small vessels.
Horton et al. (2022) J Physiol [52]	Human RCT	Healthy young adults 6 males 7 females	To compare macrovascular and microvascular functional responses to euglycemia and hyperglycaemia	Unlike meal-promoted acute hyperglycaemia, 4 h of intravenous glucose-induced hyperglycaemia enhances brachial artery flow-mediated dilatation and evokes cardiac and skeletal muscle microvascular function without impairing aortic stiffness.
Loader et al. (2017) Arterioscler Thromb Vasc Biol [53]	Human RCT	Healthy young adults (12 males) 600 mL (20 oz.) of water vs. SSB	To compare EF and VSMF.	SSB infusion mediates endothelial dysfunction with increased oxidative stress and decreasing NO bioavailability after SSB infusion.
Tabit et al. (2013) Circulation [54]	Human Prospective comparative	40 diabetics type 2 vs. 36 nondiabetic controls	To study the activity of PKCβ nuclear factor κB and reduced No	Altered eNOS activation, reduced insulin action and increased inflammatory activation. Increased PKCβ activity in endothelial insulin resistance.
Farb et al. (2016) Vasc Med [55]	Human Visceral adipose tissue arterioles	43 obese pts	To investigate the role of WNT5A-JNK leading to insulin-mediated vasodilator responses	Up-regulation of WNT5A-JNK signaling and impaired endothelial eNOS activation
Walther et al. (2015) Arterioscler Thromb Vasc Biol [56]	Human RCT	53 pts MetS without T2D vs. 25 pts T2D vs. 40 pts healthy	To compare EF and VSMF. To measure plasma glucose, insulin and inflammatory markers	MetS is associated with endothelial-dependent and endothelial-independent dysfunction, affecting both the macro- and the microvascular systems.
Bretón-Romero et al. (2016) Arterioscler Thromb Vasc Biol [57]	Human Prospective comparative	42 pts T2D vs. 43 pts healthy	To evaluate whether increased activation of Wnt5a-JNK signaling contributes to impaired EF. To determine eNOS activation and NO production	Wnt5a-induced impairment of eNOS activation and NO that was reversed by Wnt5a and JNK inhibition. Noncanonical Wnt5a signaling and JNK activity contribute to vascular insulin resistance and endothelial dysfunction.
Cho et al. (2018) Arterioscler Thromb Vasc Biol [58]	Human/Animal model	Sprague-Dawley rat vs. Human EC	To investigate whether SFRP5 could restore WNT5A-induced endothelial dysfunction in vitro and ex vivo.	Compensatory action of SFRP5 against atherosclerosis under conditions of metabolic dysfunction. SFRPS restored WNT5A-induced reduction of NO production via eNOS

Abbreviations; EC, endothelial cell; EF, endothelial function; eNOS, endothelial nitric oxide synthase; MetS, metabolic syndrome; NO, nitric oxide; PKCβ, protein kinase C-β; RCT, randomized clinical trial; SFRP5, secreted frizzled-related protein 5; SSB, sweetened beverage; T2D, type 2 diabetes mellitus; VSM, vascular smooth muscles; VSMF, vascular smooth muscle function; WNT5A-JNK, Wingless-Related Integration Site 5A-Jun N-terminal kinase; rWnt5a, recombinant Wingless-Related Integration Site 5A.

## Supplementary Materials

The following supporting information can be downloaded at: https://www.mdpi.com/article/10.3390/metabo13030430/s1, Supplementary file S1: PRISMA 2020 checklist.
